# Coordination Confined Silver‐Organic Framework for High Performance Electrochemical Deionization

**DOI:** 10.1002/advs.202401174

**Published:** 2024-05-02

**Authors:** Dun Wei, Baixue Ouyang, Yiyun Cao, Lvji Yan, Bichao Wu, Peng Chen, Tingzheng Zhang, Yuxin Jiang, Haiying Wang

**Affiliations:** ^1^ School of Metallurgy and Environment Central South University Changsha 410083 China; ^2^ College of Environmental Science and Engineering Central South University of Forestry and Technology Changsha 410004 China; ^3^ Faculty of Life Science and Technology Central South University of Forestry and Technology Changsha 410004 China; ^4^ Chinese National Engineering Research Center for Control and Treatment of Heavy Metal Pollution Central South University Changsha 410083 China

**Keywords:** capacitive deionization, cycling performance, interface stability, metal–organic framework, silver, volume change

## Abstract

Silver (Ag) is deemed a promising anode material for capacitive deionization (CDI) due to its high theoretical capacity and efficient selectivity to Cl^−^. However, the strong volume change during the conversion reaction significantly undermines the cycling performance of the Ag electrode. Additionally, achieving well‐dispersed Ag in the active matrix is challenging, as Ag electrodes prepared by conventional thermal reduction tend to agglomerate. Herein, the organic linker confinement strategy is proposed, applying metal–organic framework (MOF) chemistry between Ag nodes and organic ligands to construct Ag‐based MOF. The uniform dispersion of Ag at the molecular level, confined in the organic matrix, efficiently enhances the utilization of active sites, and strengthens the interfacial stability of Ag. Consequently, the Ag‐MOF for the CDI anode exhibits an excellent Cl^−^ removal capacity of 121.52 mg g^−1^ at 20 mA g^−1^ in 500 mg L^−1^ NaCl solution, and a high Ag utilization rate of 60.54%. After 100 cycles, a capacity retention of 96.93% is achieved. Furthermore, the Cl^−^ capture mechanism of Ag‐MOF is elucidated through density functional theory (DFT) calculations, ex situ XRD, ex situ Raman and XPS. This ingenious electrode design can offer valuable insights for the development of high‐performance conversion electrodes for CDI applications.

## Graphical abstract

Ag‐MOF prepared by the organic linker‐confined strategy with ultrafine and highly dispersed Ag nanoclusters effectively alleviates the volume expansion during electrochemical processes and improves the electrode stability.

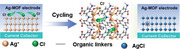

## 1 Introduction

Capacitive deionization (CDI) emerges as a promising and advanced water treatment technology,^[^ ^1^, ^2^, ^3^, ^4^, ^5^, ^6^, ^7^ ^]^ distinguished by its low energy demand,^[^ ^8^, ^9^ ^]^ high efficiency,^[^ ^10^ ^]^ and economic viability.^[^ ^11^ ^]^ This innovative approach to water treatment holds significant potential for addressing contemporary challenges through the provision of a sustainable solution, encompassing selective separation and energy recovery.^[^ ^12^, ^13^, ^14^, ^15^, ^16^, ^17^, ^18^ ^]^ The selection of electrode materials plays a pivotal role in achieving superior performance in CDI.^[^ ^19^, ^20^, ^21^, ^22^, ^23^, ^24^, ^25^, ^26^ ^]^ At present, carbon‐based electrodes reign supreme in CDI applications, primarily owing to their cost‐effectiveness,^[^ ^27^, ^28^, ^29^, ^30^ ^]^ ready availability,^[^ ^31^ ^]^ and exceptional conductivity.^[^ ^32^, ^33^, ^34^ ^]^ Nevertheless, carbon materials exhibit limitations in terms of adsorption capacity and are challenged by issues such as co‐ion exclusion effects and carbon oxidation, thus impeding the widespread implementation of CDI on a large scale.^[^ ^35^, ^36^, ^37^, ^38^ ^]^

Ag electrodes, employing faradaic conversion reactions, hold promise for CDI with their high theoretical capacity (248 mAh g^−1^), exceptional Cl^−^ selectivity, and excellent electrochemical reversibility.^[^ ^39^, ^40^, ^41^, ^42^ ^]^ The desalination mechanism of Ag in CDI involves its reaction with Cl^−^ to generate AgCl in an aqueous medium.^[^ ^43^ ^]^ In 2012, Cui et al.^[^ ^44^ ^]^ introduced the concept of a desalination battery comprising an Ag electrode for Cl^−^ capture coupled with Na_2‐x_Mn_5_O_10_, with a coulombic efficiency of up to 80%, and selective separation of Cl^−^ achieved by potential control. Subsequently, Ag electrodes have undergone thorough investigation within the realm of CDI.^[^ ^45^ ^]^ However, the strong volume change (≈225%) of Ag‐based materials during Cl^−^ conversion and release usually leads to problems of mechanical crushing, expansion of particles, and pulverization loss, which hinders the reaction kinetics and reduces the cyclic stability.^[^ ^46^, ^47^ ^]^ Combining with a conductive substrate is the most common method to improve the desalination performance of silver‐based electrodes.^[^ ^48^, ^49^, ^50^ ^]^ Yue et al.^[^ ^51^ ^]^ created a chloride storage electrode by loading reduced graphene oxide with silver (Ag@rGO), coupled with an NVO@rGO cathode. This system demonstrated a salt removal capacity of 84.2 mg g^−1^ and a charging efficiency of 94.4%. Meanwhile, Liang et al.^[^ ^52^ ^]^ synthesized Ti_3_C_2_T_x_/Ag composite electrodes with a Cl^−^ adsorption capacity of up to 135 mg g^−1^ and low energy consumption of 0.42 kWh kg^−1^ Cl^−^. Nevertheless, the inevitability of carbon oxidation in the composite electrode during prolonged cycling persists, resulting in the destabilization of the electrode.^[^ ^53^ ^]^

Metal–organic frameworks (MOFs), a novel type of crystalline porous coordination polymers created through the coordination of organic linkers and metal nodes, show great promise in ion storage applications due to their precise pore structure, design flexibility, and oxidation resistance.^[^ ^54^, ^55^, ^56^ ^]^ Constructing Ag‐based MOFs with Ag─O ligand bonds provides a solution to address the significant volume changes in Ag electrodes.^[^ ^57^, ^58^ ^]^ The coordination interactions allow homogeneous anchoring of Ag to the organic substrate at the atomic level, preventing the growth and aggregation of particles.^[^ ^59^, ^60^ ^]^ To our knowledge, the deliberate design and construction of Ag‐based MOFs through MOF chemistry to tune electrode stability has not been reported previously.

In this context, we propose an organic linker‐confined strategy that leverages metal–organic framework chemistry involving Ag nodes and organic ligands for the construction of Ag‐based MOFs. This approach facilitates the homogeneous dispersion of Ag at the molecular level, confined within an organic matrix, thereby significantly improving the utilization of active sites and enhancing the interfacial stability of Ag. As a CDI anode, the Ag‐MOF exhibited a notable Cl^−^ removal capacity of 121.52 mg g^−1^ at 20 mA g^−1^ with a NaCl concentration of 500 mg L^−1^. The Ag utilization rate reached 60.54% and the capacity retention rate up to 96.93% for 100 cycles. Furthermore, the Cl^−^ capture mechanism of the Ag‐MOF electrode was revealed by ex situ analysis combined with density functional theory (DFT). This distinctive MOF chemical design concept offers novel insights into the development of highly stable conversion electrodes for CDI.

## 2 Results and Discussion

### 2.1 Synthesis and Characterization of Ag‐MOF

**Scheme 1:**
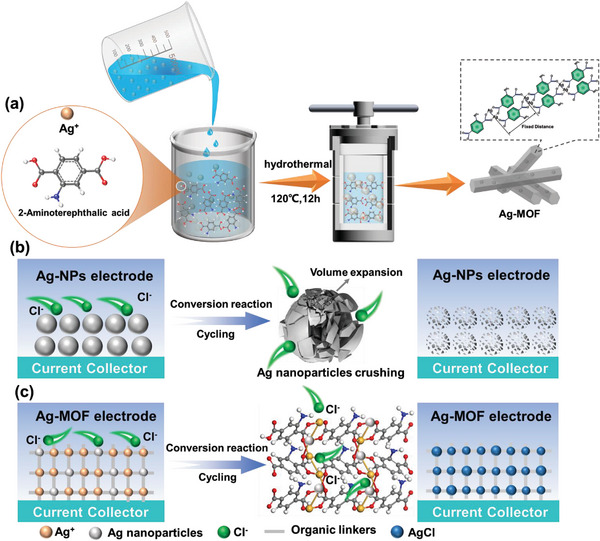
a) Schematic illustration of the synthesis of Ag‐MOF. The evolution of b) Ag‐NPs and c) Ag‐MOF electrodes during the Cl^−^ capture recycling process.

**Scheme** **1**a illustrates the facile preparation procedure of Ag‐MOF. In brief, the Ag‐MOF was synthesized by the solvothermal method based on the coordination self‐assembly reaction of 2‐aminoterephthalic acid (H2ATA) with silver nitrate, which was directly used for CDI anode without further treatment. As a comparison, the product obtained after heat treatment of Ag‐MOF, named AgNC, was also prepared. Based on previous studies,^[^ ^61^ ^]^ the Ag‐MOF possesses a 3D framework structure in which the silver ions are coordinated to three oxygen atoms of three different organic ligands and form a short Ag–Ag 2D wave‐like layer with adjacent silver by bridging carboxylic acid groups (Figure S2, Supporting Information). The 1D Ag–Ag chain formed by bridged organic ligands provides a channel for charge transfer. Scheme 1b,c reveals the structural evolution process of Ag nanoparticles (Ag‐NPs) and Ag‐MOF electrodes before and after the capture of Cl^−^ cycling. When Cl^−^ is captured, AgCl is generated on the surface of Ag‐NPs that is accompanied by a dramatic volume expansion and destroys the structure of Ag‐NPs weakening the CDI cycling performance (Scheme 1b). For Ag‐MOF electrodes, Ag atoms are confined by organic linkers and dispersed in a 3D framework structure,^[^ ^62^ ^]^ which can effectively alleviate the volume change of the Cl^−^ capture process, prevent the agglomeration of the active species, and increase the electrochemical reversible capacity (Scheme 1c).

**Figure 1:**
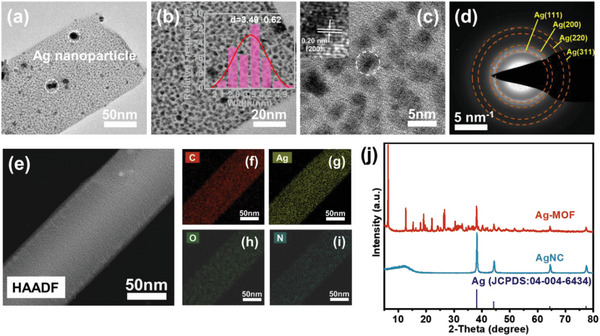
Morphological characterization of Ag‐MOF: a,b) TEM and corresponding the size distribution statistical histograms in inset b, and c) HRTEM images, d) selected area electron diffraction, e–i) elemental mappings of C, Ag, O, N, and j) XRD pattern.

Scanning electron microscopy (SEM) and transmission electron microscopy (TEM) were performed to reveal the structure and microscopic morphology of the materials. Ag‐MOF presents a typical nanorod‐like morphology with a smooth surface (Figure S3a, Supporting Information). After pyrolysis, the surface of the obtained AgNC was rough (Figure S3b,c, Supporting Information). The TEM image results show that the ultrafine Ag nanoclusters with an average size of 3.49 nm are homogeneously distributed on the Ag‐MOF (**Figure** **1**a,b). The ultrafine particle size of nano‐Ag can promote the reaction kinetics and increase the utilization of active components in the CDI process. In addition, some Ag nanoparticles were also found in Ag‐MOF, which were due to the partial reduction of silver ions in the solvothermal reaction. (Figure 1a). At the same time, the Ag content in Ag‐MOF and AgNC was analyzed by TG curves (Figure S4, Supporting Information). A clear lattice fringe with a spacing of 0.20 nm was observed in the high‐resolution TEM (HRTEM) image, corresponding to the (200) crystalline plane of metal Ag. (Figure 1c). However, the Ag particle size in the AgNC product significantly enlarged to 9.42 nm after Ag‐MOF pyrolysis due to carbothermal reduction (Figure S5, Supporting Information). In addition, the selected area electron diffraction (SAED) pattern displayed a set of diffraction rings that confirm the presence of the metal Ag^0^ (Figure 1d). The high‐angle annular dark‐field scanning TEM (HAADF‐STEM) and elemental mapping revealed the homogeneous distribution of C, Ag, O, and N elements in Ag‐MOF (Figure 1e–i). The X‐ray diffraction patterns (XRD) of the samples are shown in Figure 1j. The XRD pattern of AgNC shows four diffraction peaks that index the crystal structure of face‐centered‐cubic silver (JCPDS 04‐004‐6434). The XRD results of Ag‐MOF were similar to those reported before (CCDC 198 096), and the phase of the metal Ag was also indexed.

**Figure 2:**
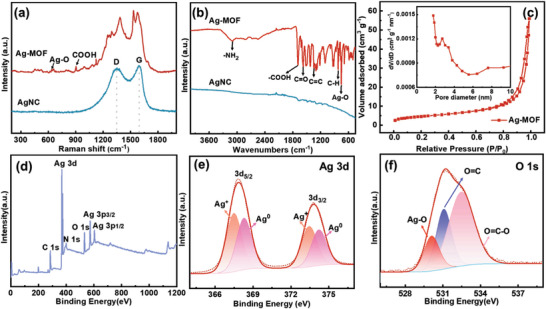
a) Raman spectra and b) FTIR spectra for Ag‐MOF and AgNC. c) N_2_ adsorption–desorption isotherms, inset is the pore size distribution curve, d) XPS survey spectra, e) high‐resolution Ag 3d and f) O 1s spectra for Ag‐MOF.

The physicochemical properties and compositions of the obtained Ag‐MOF and AgNC materials were further investigated. The Raman spectra of AgNC showed clear D‐band (1350 cm^−1^) and G‐band (1588 cm^−1^), assigned to defective carbon and graphitic carbon (**Figure** **2**a), respectively, indicating the organic ligand pyrolysis of Ag‐MOF converted to carbon. Especially, the Ag─O stretching vibration located at 652 cm^−1^ was detected,^[^ ^63^ ^]^ confirming the mutual interaction of Ag^+^ and the carboxyl functional group of the organic linker, which can also be attested by FTIR spectra (Figure 2b). In the FTIR spectra, several intense absorption bands at 3497, 1680, 1574, 1348, 823, and 757 cm^−1^, corresponding to the bending vibration of the ─NH_2_ group, ─COOH, C═O group stretching vibration, and Ag─O vibration, respectively.^[^ ^57^ ^]^ After heat treatment, functional groups disappear due to the decomposition of organic ligands. The porosity of the as‐prepared materials was investigated by N_2_ adsorption–desorption, and the relevant results are shown in Figure 2c, Figure S6, and Table S1 (Supporting Information). The adsorption isotherm of Ag‐MOF shows type IV with a characteristic hysteresis loop at high relative pressures, which confirms the mesoporous feature.^[^ ^64^ ^]^ The BET specific surface area of Ag‐MOF was 85.23 m^2^ g^−1^, and the pore size was mainly distributed between 2–4 nm. The macropores of Ag‐MOF determined by mercury porosimetry were mainly distributed at 100 nm (Figure S6a, Supporting Information). The abundant mesopores and macropores facilitate electrolyte penetration and ion diffusion.

X‐ray photoelectron spectroscopy (XPS) was performed to investigate the surface chemical state of the samples (Figure 2d–f; Figure S7,S8, and Table S2, Supporting Information). The XPS survey spectra of Ag‐MOF exhibited the presence of C, Ag, O, and N elements (Figure 2d), which aligns with the TEM‐mapping results. The high‐resolution Ag 3d spectra showed a pair of peaks, the binding energies located at 367.46 and 373.47 eV corresponding to the Ag 3d_5/2_ and Ag 3d_3/2_ peaks of Ag^+^, and the peaks at 368.26 and 374.25 eV are attributed to the Ag 3d_5/2_ and Ag 3d_3/2_ binding energies of the metal Ag^0^ (Figure 2e).^[^ ^65^ ^]^ In contrast, AgNC shows only the presence of metal Ag^0^, which is due to the complete reduction of Ag^+^ to Ag nanoparticles by carbothermal reduction (Figure S8b, Supporting Information). The high‐resolution O 1s spectra were deconvoluted into three peaks, corresponding to the Ag─O (530.13 eV) peak, the C═O (531.10 eV) peak, and the O═C─O (532.51 eV) peak (Figure 2f).^[^ ^66^ ^]^ The presence of an Ag─O bond further confirms the coordination of Ag^+^ with the carboxyl functional group of the organic ligand, which is in accordance with the Raman and FTIR results.

### 2.2 Evaluation of Electrochemical Performance

**Figure 3:**
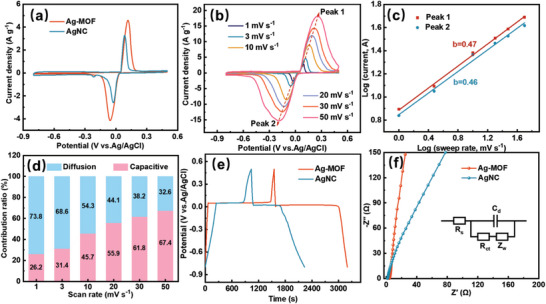
a) CV curves at 1 mV s^−1^ for Ag‐MOF and AgNC, b) CV curves at different scan rates, d) b‐value calculations, and e) capacity contributions at different scan rates for Ag‐MOF electrode. c) GCD curves at 100 mA g^−1^, and f) Nyquist plots of Ag‐MOF and AgNC electrodes (inset is equivalent circuit diagram).

A three‐electrode system was used to investigate the electrochemical properties of Ag‐MOF and AgNC in a 1 m NaCl electrolyte. First, the cyclic voltammetry (CV) curves of Ag‐MOF and AgNC electrodes were measured at 1 mV s^−1^ (**Figure** **3**a). The CV curves for all electrodes exhibited a distinct oxidation peak at 0.12 V and a reduction peak at −0.05 V (vs Ag/AgCl), relating to the reversible electrochemical conversion between Ag and AgCl. The CV curves of AC showed a rectangular‐like shape without redox peaks, indicating the ion capture based on the electric double‐layer capacitance (Figure S9a,b, Supporting Information). Ag‐MOF presented a much higher current density than AgNC, indicating a high capacity. Based on the integrated area of the CV curve, the Ag‐MOF exhibited a larger specific capacitance of 150.96 F g^−1^ than the AgNC electrode (110.13 F g^−1^). The CV curves at different scan rates were further tested, and the Ag‐MOF electrodes all presented the highest specific capacitance (Figure 3b; Figure S9c,f, Supporting Information). The enhanced capacity of Ag‐MOF is achieved through the full utilization of highly dispersed ultrafine Ag nanoclusters, completely unlocking the chlorine storage potential of the active sites.

Based on CV tests at different scan rates, the dechlorination kinetic processes of Ag‐MOF and AgNC were further investigated (Figure 3b). The correlation between redox peak current (i, A) and scan rate (v, mV s^−1^) follows the power law relation proposed by Bruce Dunn:^[^ ^67^ ^]^

(1) $$i=av^{b}$$

(2) $$\log\left(i\right)=b\log\left(v\right)+\log\left(a\right)$$

where b is determined by the slope of the log(i) versus log(v) plot, which is an important parameter to evaluate the dechlorination mechanism. Generally, the b‐value is approaching 0.5 for the diffusion‐controlled process and the b‐value is close to 1 for the capacitive behavior. The calculated b‐values of Peak 1 and Peak 2 for Ag‐MOF are 0.47 and 0.46, respectively, indicating that the electrochemical dechlorination mechanism of Ag‐MOF is mainly a diffusion‐controlled process (Figure 3c). The b‐values calculated for AgNC electrodes reveal similar results (Figure S9d, Supporting Information). The contribution of capacity can be calculated according to the method proposed by Dunn:^[^ ^68^ ^]^

(3) $$i=k_{1}v+k_{2}v^{1/2}$$

where k_1_v and k_2_v^1/2^ denote the capacitive contribution and the diffusion control contribution, respectively. The calculated results are shown in Figure 3d, and the electrochemical reaction of Ag‐MOF at a low scanning rate is mainly a diffusion control process. The diffusion control contribution of Ag‐MOF is 73.8% at the scan rate of 1 mV s^−1^, significantly higher than that of AgNC (57.7%, Figure S9e, Supporting Information). This result confirms that more Ag in Ag‐MOF participates in the Cl^−^ capture reaction, meaning a high Ag utilizing rate.

The electrochemical performance of the as‐prepared electrodes was evaluated by the galvanostatic charge‐discharge (GCD) method with a cutoff potential ranging from −0.8 to 0.5 V (vs Ag/AgCl). As shown in Figure 3e, the GCD curves of all electrodes possessed a charging and discharging platform at a potential of 0 V (vs Ag/AgCl), confirming further that the Cl^−^ capture process is a battery‐type faradaic reaction.^[^ ^49^ ^]^ The Ag‐MOF all presented the longest discharge time at different current densities as compared to AgNC, indicating that Ag‐MOF with excellent GCD specific capacity (Figure S10a–c, Supporting Information). The electrochemical impedance spectra (EIS) displayed in Figure 3f, and the equivalent circuit fitting results presented in Table S3 (Supporting Information). The solution resistances (*R*_s_, intersection with the real axis) of Ag‐MOF and AgNC were 3.95 and 1.83 Ω, respectively, confirming the good solution accessibility of the prepared electrodes. The EIS curves of Ag‐MOF showed a small semicircle, confirming their low charge transfer resistance (*R*_ct_, 1.85Ω) compared to AgNC (1.97 Ω). The as‐prepared electrodes all showed low charge transfer resistance (*R*_ct_), indicating the potential advantages of faradaic electrodes. Moreover, the Nyquist plots of Ag‐MOF under the low‐frequency region exhibit higher slopes than that of AgNC, and the result proves the faster ion diffusion process of Ag‐MOF due to the ordered structure of MOFs.

### 2.3 HCDI (Hybrid Capacitive Deionization) Performance

**Figure 4:**
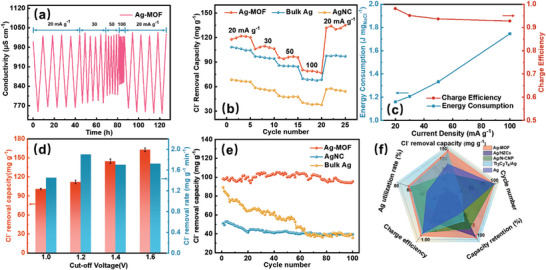
a) Conductivity profile of Ag‐MOF in 500 mg L^−1^ NaCl for 5 electrosorption cycles at different current densities, b) rate performance of Ag‐MOF, AgNC, and bulk Ag electrodes at different current densities, c) energy consumption and charge efficiency at different current densities, d) Cl^−^ removal capacity and removal rate at different voltage windows for Ag‐MOF electrodes, e) The 100 cycles performance in 50 mA g^−1^ with 500 mg L^−1^ NaCl solution for Ag‐MOF, AgNC and bulk Ag electrodes, f) Performance comparison of Ag‐MOF anodes with previously reported Ag‐based electrodes.^[^ ^45^, ^48^, ^52^, ^69^ ^]^

The hybrid capacitive deionization (HCDI) system was assembled with an Ag‐based electrode and activated carbon (AC) as anode and cathode, respectively, and the deionization performance of all samples was examined in constant current mode. Initially, the CV curves of two CDI systems, Ag‐MOF//AC and AC//AC, were determined (Figure S11a,b, Supporting Information). The CV curves of the Ag‐MOF//AC‐based hybrid CDI system showed distinct redox peaks, indicating that the hybrid CDI system captured ions by Faradaic reactions. In contrast, the CV curves of the CDI system for AC//AC show a rectangular shape, indicating the presence of electric doubled‐layer capacitance. In the deionization process, Cl^−^ ions were captured by the Ag‐based electrode, while Na^+^ ions were adsorbed onto the AC electrode. This resulted in a decrease in conductivity with increasing voltage, and a reverse phenomenon during salinization, revealing the reversible adsorption–desorption process of salt ions (**Figure** **4**a; Figure S11g, Supporting Information). Based on the conductivity‐concentration relationship (Figures S1 and S11c,d, Supporting Information), the Cl^−^ removal capacity of Ag‐MOF, AgNC, and bulk Ag was calculated for repeated desalination tests of 5 cycles at different current densities. The Ag‐MOF electrode showed the optimal Cl^−^ removal capacity over AgNC and bulk Ag at all current densities (Figure 4b). Notably, the Ag‐MOF achieved a Cl^−^ removal capacity of up to 121.52 mg g^−1^ at 20 mA g^−1^ with 500 mg L^−1^ NaCl. The fast ion removal rate was realized at higher current densities, and the Cl^−^ removal rate of Ag‐MOF reached a maximum value of 2.6 mg g^−1^ min^−1^ at 100 mA g^−1^ (Figure S11e, Supporting Information). In addition, the Ag utilization rate of Ag‐MOF electrodes reached as much as 60.54% compared to AgNC (24.74%) and Bulk Ag (32.61%) electrodes (Figure S11f, Supporting Information). The energy consumption of the Ag‐MOF‐based HCDI system at a low current density of 20 mA g^−1^ was 1.16 J mol^−1^ due to sufficient ion diffusion time, and the charge efficiencies are all higher than 0.9 at different current densities (Figure 4c).

The HCDI performance of Ag‐MOF at different cut‐off voltages and different NaCl concentrations was systematically investigated. Figure 4d shows the effect of cut‐off voltage on the electro‐sorption performance of Ag‐MOF in 500 mg L^−1^ NaCl solution at 50 mA g^−1^, and the corresponding conductivity profile is displayed in Figure S11h (Supporting Information). The high cut‐off voltage realized high Cl^−^ removal capacity. The Cl^−^ removal capacity of Ag‐MOF increases from 100.83 to 162.68 mg g^−1^ as the voltage window increases from 1.0 to 1.6 V. The highest Cl^−^ removal rate of 1.9 mg g^−1^ min^−1^ was obtained at a cut‐off voltage of 1.2 V. Nonetheless, the high cut‐off voltage results in severe energy consumption, which increases from 0.92 (−1.0–1.0 V) to 1.88 J mol^−1^ (−1.6–1.6 V, Figure S11i, Supporting Information). Meanwhile, the Cl^−^ removal capacity of the Ag‐MOF also increased gradually with the increase of the initial concentration of NaCl, which was from 95.84 (500 mg L^−1^) to 136.78 (3000 mg L^−1^) at 50 mA g^−1^ (Figure S11j, Supporting Information). Moreover, the deionization performance of Ag‐MOF was also investigated at a constant voltage of 1.2 V. The Ag‐MOF‐based HCDI system showed a NaCl removal capacity of 115.16 mg L^−1^ with a maximum salt removal rate of up to 12.04 mg g^−1^ min^−1^ in 500 mg L^−1^ NaCl solution at 1.2 V constant voltage (Figure S12a–d, Supporting Information). In particular, the prepared Ag‐MOF electrodes show great application promise compared to the previously reported Ag, Bi‐based CDI electrodes (Figure S12e and Table S4, Supporting Information).

Cycling stability is an essential parameter for evaluating electrode materials for practical applications. In this investigation, the cycling performance of Ag‐MOF, AgNC, and Bulk Ag was determined for 100 cycles in a 500 mg L^−1^ NaCl solution at 50 mA g^−1^. As shown in Figure 4e, the dechlorination performance of AgNC and bulk Ag electrodes gradually decayed over 100 cycles with final capacity retention of 76.92% and 40.26%, respectively. Especially, the Cl^−^ removal performance of the bulk Ag electrode significantly declined during the initial 80 cycles, which can be attributed to the volume expansion occurring in the cycling process resulting in a loss of active components. For the Ag‐MOF electrode, the Cl^−^ removal capacity after 100 cycles was 95.41 mg g^−1^ with capacity retention as high as 96.93%, and the charging efficiency remained at 0.86 (Figure S13, Supporting Information). The Ag‐MOF electrode showed a significant increase in reversible capacity and HCDI cyclability compared to the control electrode. Further, the CDI performance of the as‐prepared Ag‐MOF electrode and the currently reported Ag‐based electrodes were also comparatively analyzed using radar charts (Figure 4f). The Ag‐MOF‐based HCDI system showed excellent performance in terms of Ag utilization rate, Cl^−^ removal capacity, cycle number, capacity retention, and charge efficiency, confirming the great application prospects.

### 2.4 Mechanism Investigation

**Figure 5:**
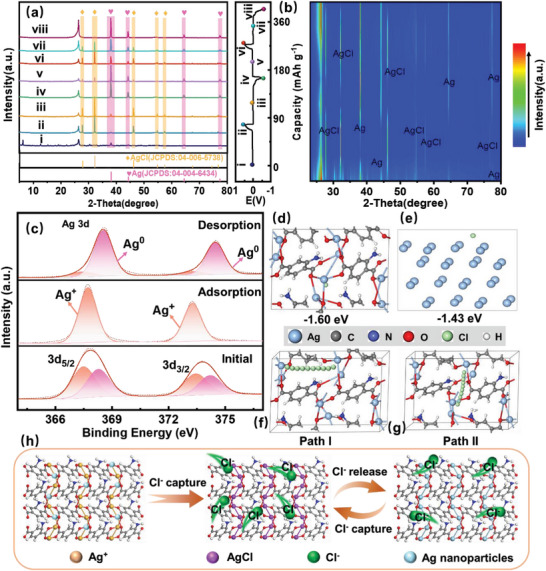
a) XRD pattern of Ag‐MOF electrode under different charge/discharge states, b) corresponding contour maps. c) The change of Ag 3d spectra before and after electrosorption and under electrodesorption for Ag‐MOF electrode. d,e) Optimized adsorption configurations of Ag‐MOF and metal Ag (111) with one Cl ion. f,g) Schematic diagram of the two diffusion paths of Cl ions on Ag‐MOF clusters. h) Schematic illustration of the mechanism of reversible Cl^−^ capture in Ag‐MOF.

The Cl^−^ capture mechanism of Ag‐MOF with organic ligand‐confined highly dispersed ultrafine Ag nanoclusters is analyzed in detail. At first, the XRD of the Ag‐MOF electrode in NaCl solution without applied voltage was performed and the phase was almost completely converted to AgCl, confirming the presence of Ag^+^ in the Ag‐MOF structure (Figure S14, Supporting Information). The phase transitions of Ag‐MOF electrodes under different charging and discharging states were characterized based on ex situ XRD. In the original state, the Ag‐MOF electrode detected only the peak of the metal Ag^0^ (**Figure** **5**a(i)). After the first complete charging, the Ag peak disappears entirely, and metal Ag^0^ and Ag^+^ in the Ag‐MOF structure are completely converted to AgCl. (Figure 5a(ii)). Subsequently, the phase of Ag^0^ reappeared and coexisted with AgCl within the electrode after partial discharge of the electrode (Figure 5a(iii)). Eventually, the peak of AgCl in the electrode material vanished after the first complete discharge that converted into the metal Ag^0^ phase (Figure 5(iv)). Thus, the conversion reaction between Ag^+^, Ag^0,^ and AgCl occurs during the first charge/discharge process (Ag^+^ + Cl^−^ →AgCl, Ag + Cl^−^ ⇌ AgCl + e^−^). In the subsequent electrochemical process, the electrode material undergoes only a reversible conversion reaction between Ag^0^ and AgCl (Figure 5a(v–viii)), which is more vividly revealed by the contour maps of the corresponding XRD patterns (Figure 5b). The XPS spectra of Ag 3d before and after the electro‐sorption of Ag‐MOF electrode were analyzed (Figure 5c). In the initial state, the Ag 3d of the Ag‐MOF structure XPS spectra were fitted with two pairs of peaks, corresponding to Ag^+^ (367.46 and 373.47 eV) and Ag^0^ (368.26 and 374.25 eV). However, the Ag 3d XPS spectra of the electrode material only detect two peaks corresponding to Ag 3d_3/2_ and Ag 3d_5/2_ of Ag^+^ after capturing Cl^−^, further confirming that the active Ag species in the electrode material was completely converted to AgCl. Subsequently, the Cl^−^ was released from the electrode structure and AgCl was converted to Ag^0^ with only a small amount of Ag^+^ present, which was consistent with the XRD analysis of the different charging and discharging states. Meanwhile, the Raman spectra of Ag‐MOF electrode under different charging and discharging states can also further demonstrate the reversible capture process of Cl^−^ (Figure S15, Supporting Information).

The density functional theory (DFT) calculations were conducted to further understand the excellent Cl^−^ capture performance of Ag‐MOF electrodes. The two models of Ag‐MOF and metal Ag (111) were constructed to explore the adsorption energy of chloride on the electrode surface (Figure 5d,e). The adsorption energy of Ag‐MOF was calculated to be −1.60 eV, as more negative than that of metal Ag (111) (−1.43 eV), indicating the strong Cl^−^ capture behavior of Ag‐MOF. The diffusion process of Cl^−^ in the Ag‐MOF structure is also analyzed and the diffusion barriers of Cl^−^ in different paths are calculated by DFT. As shown in Figure 5f,g, two diffusion pathways were established: a) diffusion of Cl^−^ ions between Ag in neighboring clusters (Path I), and b) diffusion of Cl^−^ ions between neighboring Ag in the same cluster (Path II). As shown in Figure S16 (Supporting Information), the Cl^−^ possesses the lowest diffusion barrier along path II, which indicates that path II is most favorable for Cl^−^ diffusion. Therefore, Ag‐MOF exhibits high desalination performance due to stronger Cl^−^ adsorption energy and excellent Cl^−^ diffusion process.

Based on the above analysis, the Cl^−^ storage mechanism of Ag‐MOF can be proposed. In the first adsorption process, the active Ag^+^ in the Ag‐MOF structure reacts directly with Cl^−^ to form AgCl even in the absence of an electric field. At the same time, the metal Ag^0^ in Ag‐MOF also reacted with Cl^−^ to convert to AgCl under the electric field. Subsequently, the reversible conversion reaction between AgCl and Ag in the Ag‐MOF structure under the electric field realizes the release and capture of Cl^−^ (Figure 5h). The advantages of Ag‐MOF as a Cl^−^ capture electrode can be attributed to the following points: i) The ordered porous structure of Ag‐MOF facilitates the transport of ions, and disperses the stress caused by the conversion reaction. ii) The ultrafine and highly dispersed Ag nanoclusters strengthened the reaction efficiency and alleviated the volume expansion. iii) The coordination structure of the organic linker with the metal ion restricts the migratory agglomeration of Ag and reinforces the stability.

## 3 Conclusion

The Ag‐MOF material with an organic ligand confinement structure was successfully designed by a facile solvothermal method for high‐performance CDI anodes. The intrinsic ordered porous structure of MOF can promote ion diffusion. The highly dispersed ultrafine Ag nanoclusters can enhance the utilization efficiency of active sites and alleviate volume expansion. The organic linker coordination structure strengthens the interfacial stability of the metal centers and restricts the migratory agglomeration of the active sites during electrochemical processes. When applied as a Cl^−^ selective capture electrode coupled with an AC cathode for HCDI, the Ag‐MOF provided an excellent Cl^−^ removal capacity of 121.52 mg g^−1^ at 20 mA g^−1^ with 500 mg L^−1^ NaCl, and the Ag utilization rate was as high as 60.54%. Moreover, the Ag‐MOF electrode also delivered excellent cycling performance, with a capacity retention of 96.93% at 50 mA g^−1^ for 100 cycles. In addition, the Cl^−^ diffusion process and capture mechanism of Ag‐MOF were detailed investigated by DFT calculations, ex situ XRD, ex situ Raman, and XPS. This work can provide a novel insight and strategy for designing high‐performance Cl^−^ conversion electrodes for CDI.

## 4 Experimental Section

### Synthesis of the Ag‐MOF

The Ag‐MOF was prepared by a simple solvothermal method. First, 1 mmol AgNO_3_ was dissolved in 50 mL of anhydrous methanol (solution A), and 1 mmol 2‐aminoterephthalic acid was dissolved in 10 mL DMF solution (solution B), then, solution B was slowly added dropwise to solution A by using a syringe pump. Subsequently, the mixture was transferred to a 100 mL Teflon‐lined steel reactor, sealed, and heated at 120 °C for 12 h. The resulting precipitate was collected by centrifugation, washed three times alternately with DMF and ethanol, respectively, and dried overnight at 60 °C in a vacuum oven.

### Synthesis of the AgNC

The AgNC was prepared by pyrolysis of the Ag‐MOF precursor. Briefly, the as‐prepared Ag‐MOF was placed in a tube furnace and heated to 800 °C for 2 h at a heating rate of 5 °C min^−1^ in Ar. After cooling naturally to room temperature, the black product is AgNC.

Materials and chemicals, characterization methods, electrochemical measurements, and CDI experiments are provided in detail in the Supporting Information.

### Statistical Analysis

The electrochemical performances, such as CV, GCD, and EIS, were all utilized directly from the original data of the measurements (without any pre‐processing) by employing the electrochemical workstation CHI760E (CH Instruments, Inc., America). Conductivity is directly using the original data measured by the conductivity meter DDSJ‐308F (INESA, China). Raman data were normalized and analyzed by OriginPro software. All data plotting was carried out using OriginPro software (Origin Lab, America).

## Conflict of Interest

The authors declare no conflict of interest.

## Supporting information

Supporting Information

## Data Availability

The data that support the findings of this study are available in the supplementary material of this article.
