# Supplementary material for: Coordination Confined Silver‐Organic Framework for High Performance Electrochemical Deionization
Source: Adv Sci (Weinh). 2024 May 2;11(28):2401174. doi: 10.1002/advs.202401174 (PMC11267271; doi:10.1002/advs.202401174)
Supplement: Supplementary file 1 — Supporting Information [file ADVS-11-2401174-s001.pdf]

ADVANCED SCIENCE
Open Access

Supporting Information

for *Adv. Sci.*, DOI 10.1002/advs.202401174

Coordination Confined Silver-Organic Framework for High Performance Electrochemical Deionization

Dun Wei, Baixue Ouyang, Yiyun Cao, Lvji Yan, Bichao Wu, Peng Chen, Tingzheng Zhang, Yuxin Jiang and Haiying Wang**

Supporting Information

Coordination Confined Silver-Organic Framework for High Performance Electrochemical Deionization

Dun Wei, [a] *Baixue Ouyang,* [a] *Yiyun Cao,* [a] *Lvji Yan* [a], *Bichao Wu* [a], *Peng Chen,* [a] *Tingzheng Zhang,* [a] *Yuxin Jiang,* [c,] * *Haiying Wang* [a,b,*]

[a] *School of Metallurgy and Environment, Central South University, Changsha 410083, China*

[b] *Chinese National Engineering Research Center for Control and Treatment of Heavy Metal Pollution, Central South University, Changsha 410083, China*

[c] *College of Environmental Science and Engineering, Central South University of Forestry and Technology, Changsha 410004, China*

*To whom correspondence should be addressed. Email: jiangyuxin@csuft.edu.cn, haiyw25@yahoo.com.

The Supporting Information includes 21 Pages, 16 Figures and 4 Tables.

Table of Contents

Figure S1………...S7

Figure S2………...S7

Figure S3………...S8

Figure S4……………..S8

Figure S5……………..S9

Figure S6……………..S9

Figure S7……………..S10

Figure S8……………..S11

Figure S9……………..S12

Figure S10…………........................... ....S13

Figure S11…………………………………………………………......................S14

Figure S12………………………........……………………....………....…...S15

Figure S13………………………....………......……………………....………........S15

Figure S14……………………………………………………………...........……S16

Figure S15……………………………………………………………...........……S17

Figure S16……………………………………………………………...........……S17

Table S1……………………………………………………………………………S18

Table S2..……………………………………………………………………………S18

Table S3..……………………………………………………………….............S18

Table S4..………………………………………………………………....................S19

Materials and Chemicals.

Silver nitrate ($AgNO_3$), 2-aminoterephthalic acid, 1-methyl-2-pyrrolidone (NMP, 99.5%) were purchased from Aladdin Reagent Co., Ltd. Anhydrous methanol (CH_3OH),

sodium chloride (NaCl), N, N-dimethylformamide (DMF) and Polyvinylidene fluoride (PVDF) were purchased from Shanghai Maclean Biochemical Technology Co., Ltd. All reagents were used directly without further purification. Commercial activated carbon (AC, XFP01) was purchased from Nanjing XFNANO Materials Tech Co., Ltd.

Characterization Methods.

The morphology and microstructure of the samples were observed by using field emission scanning electron microscopy (FESEM, LEOL JSM-7900F) and transmission electron microscopy (TEM, JEOL JEM-2100). Powder X-ray diffraction (XRD, Bruker, Advance D8, Switzerland) patterns were used to analyze the phase structure of the sample. X-ray Photoelectron Spectroscopy (XPS, Thermo Scientific K-Alpha) was employed to study the chemical composition of the surfaces of the samples. Raman Spectroscopy was recorded by laser micro confocal Raman spectroscopy (Renishaw, inVia) at a wavelength of 532 nm. Fourier transform infrared spectrometer (FTIR, Thermofisher Scientific, Nicoket iS50) was tested to analyze the functional groups on the sample surface by ATR mode. The nitrogen sorption-desorption isotherms of the samples were measured on a Surface Area and Porosimeter System (ASAP 2020 Micromeritics sorption analyzer). Thermo-analysis (TG) was experimented with to study the weight-loss behaviors by employing a TG analyzer (TGA5500) from room temperature to 900 °C at a heating rate of 10 °C min^{-1} in Air.

Electrochemical Measurements.

The electrochemical properties of all the samples were determined in a three-electrode system with an electrolyte of 1 M NaCl solution. Conductive carbon paper

coated with active material (area 1×1 cm^2, mass 4 mg) was used as the working electrode, Ag/AgCl electrode and platinum sheet electrode were used as reference and counter electrodes, respectively. Cyclic voltammetry (CV), galvanostatic charge-discharge (GCD) and electrochemical impedance spectroscopy (EIS) were analyzed by an electrochemical workstation (CHI 760E). EIS recording in the frequency range of 0.01 Hz-100 kHz.

The specific capacitance (C_s, F g^{-1}) of the electrode calculated from the CV curve is as follows equation (S1):

$$C_s = \frac{\int idV}{2mv\Delta V} \quad \text{(S1)}$$

Where i, m, v and ΔV represent the response current (A), mass of active material (g), scan rate (V s^{-1}) and voltage window (V), respectively.

The specific capacitance (C_v, mAh g^{-1}) of the electrode based on the GCD curve is calculated by the following equation:

$$C_v = \frac{I\Delta t}{m} \quad \text{(S2)}$$

Where I is the response current (A), Δt is the discharge time (h), and m is the mass of the electrode active material (g).

CDI Experiments.

The CDI experiments were carried out in a batch mode recirculation system. The electrodes were prepared as follows: a homogeneous slurry mixture of active material, conductive carbon black and PVDF was prepared in the ratio of 8:1:1 by weight. Subsequently, the slurry was coated on the surface of the titanium plate and vacuum dried at 60°C for 12 h. The effective area of the electrode plate is 5.0×5.0 cm^2 and the

final mass loading is 45 mg. A volume of NaCl solution is pumped into the CDI unit for circulation using a peristaltic pump. The deionization experiments were powered by a battery test system (Land, CT2001A) and operated in constant current mode. The NaCl solution conductivity is monitored and recorded in real-time by an online conductivity meter (INESA, DDSJ-308F). Moreover, the relationship curves of NaCl concentration and conductivity are provided in **Figure S1**. The Cl^- removal capacity, Cl^- removal rate, charge consumption, charge efficiency and energy consumption related formulas are provided in equations S3-S7, respectively.

The Cl^- removal capacity (Γ, mg g^{-1}) is calculated according to the following equation:

$$\Gamma = \frac{(C_0 - C_t) \times V \times M_{Cl^-}}{m \times M_{NaCl}} \tag{S4}$$

Where C_0 (mg L^{-1}), C_t (mg L^{-1}), V (L) and m (g) are the initial concentration, final concentration, volume of NaCl solution and mass of anode material, respectively. M_{Cl-} (35.5 g mol^{-1}) and M_{NaCl} (58.5 g mol^{-1}) are the molar mass of Cl^- and NaCl, respectively.

The Cl^- removal rate (Φ, mg g min^{-1}) was calculated on the basis of the following equation:

$$\Phi = \frac{\Gamma \times 60}{t} \tag{S5}$$

Where t (s) is the desalination time.

The charge efficiency (Λ), and energy consumption (E_c, J mg_{Cl}-^{1}) are calculated based on the following equations:

$$\Sigma = \frac{It}{m} \tag{S6}$$

$$\Lambda = \frac{\Gamma \times F}{M_{Cl^-} \times \Sigma \times 1000} \tag{S7}$$

$$E_c = \frac{i \int U dt}{(C_0 - C_t) \times V} \tag{S8}$$

Where U is the voltage (V) in the desalination process and F is the Faraday constant (96485 C mol^{-1}).

The Ag utilization rate is calculated as follows:

$$\mu_{Ag} = \frac{\Gamma}{328.64 \times W_{Ag}\%} \tag{S9}$$

Where 328.64 mg is the theoretical Cl^- adsorption capacity of 1 g of Ag, $W_{Ag}\%$ is the content of Ag in the electrode.

Computational methods

The calculations are performed by means of first-principles calculations in CASTEP. In the model optimization process, the generalized gradient approximation (GGA) with the Perdew-Burke-Ernzerhof (PBE) formulation is used to describe the exchange-correlation potential. The ground state of the supercells was determined using the Broyden-Fletcher-Goldfarb-Shanno (BFGS) method, with convergence criteria set at an energy change of less than 10^{-5} eV per atom, a force below 0.02 eV $Å^{-1}$, stress less than 0.05 GPa, and displacement change less than 0.001 Å. The cutoff energy is set to 450 eV. The diffusion energy barrier of Cl^- is obtained by the linear/quadratic synchronous transport (LST/QST) method.

The adsorption energy of Cl is calculated by the following equation:

$$\Delta \text{E} = E_{Cl-X} - E_X - E_{Cl} \tag{S9}$$

where E(eV) denotes the final energy of the optimized model; X denotes Ag-MOF or Ag.

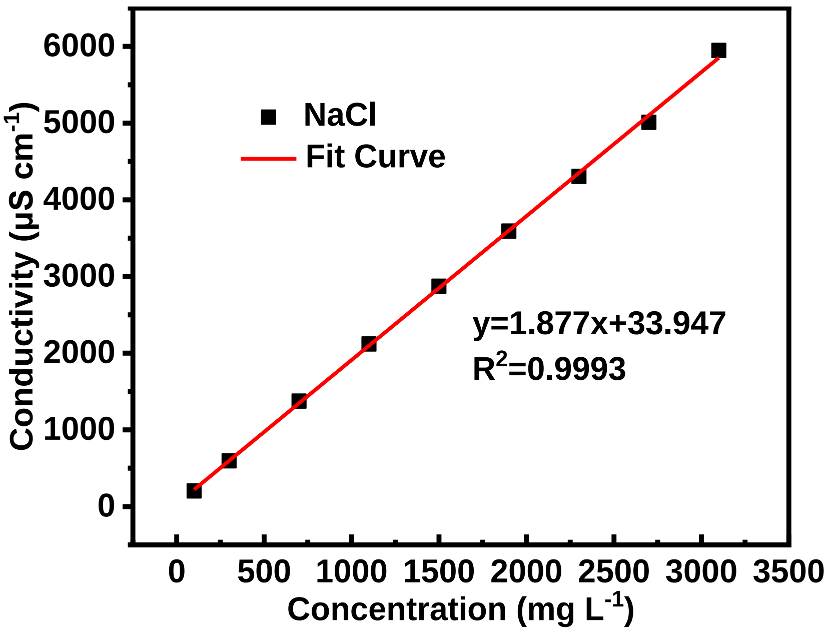

Figure S1. The standard curve of the relationship between concentration (mg L^{-1}) and conductivity (μS cm^{-1}) of NaCl solution

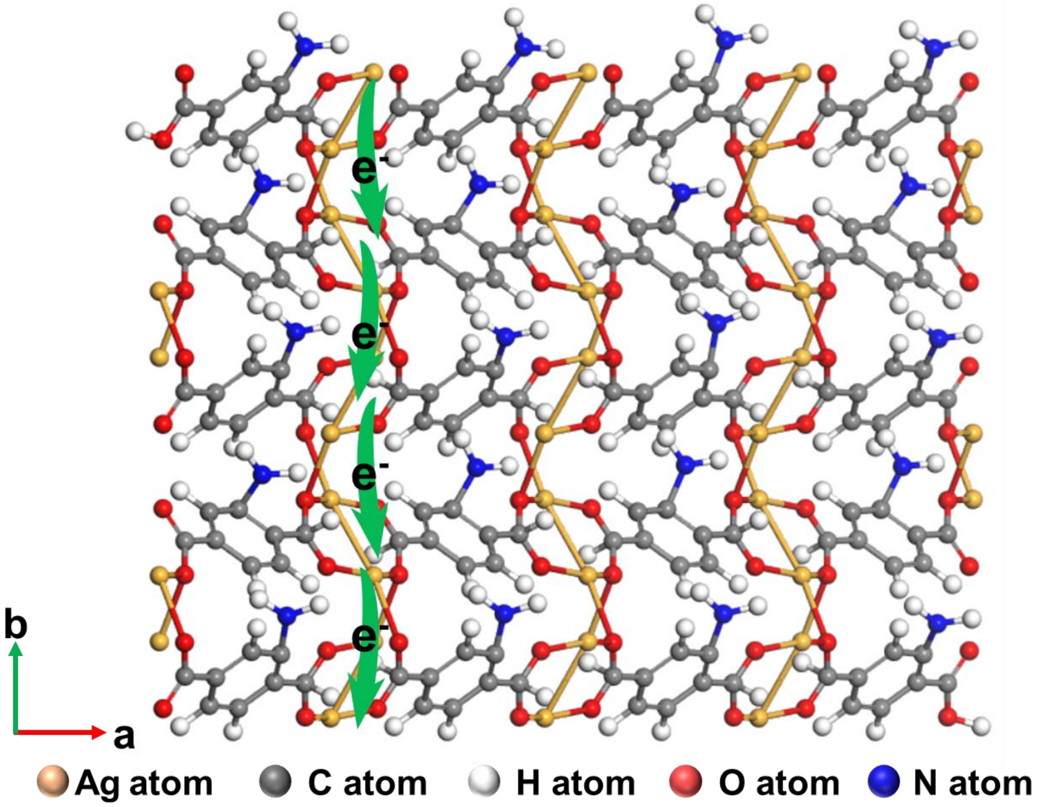

Figure S2. Crystal structure of the Ag-MOF.

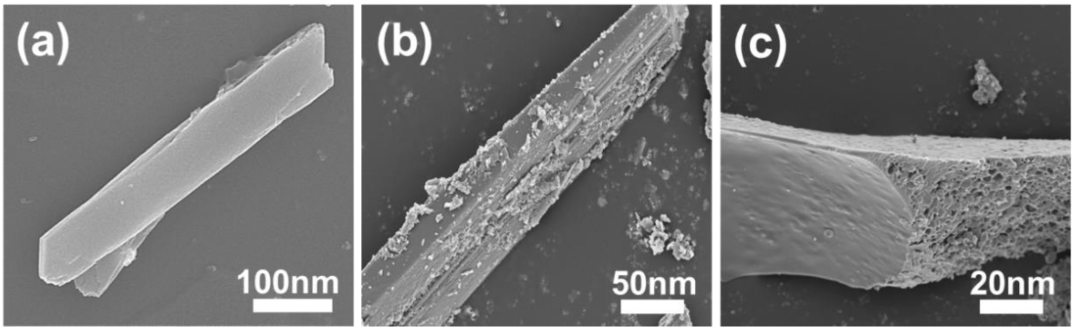

Figure S3. The SEM image of (a) Ag-MOF, (b-c) AgNC.

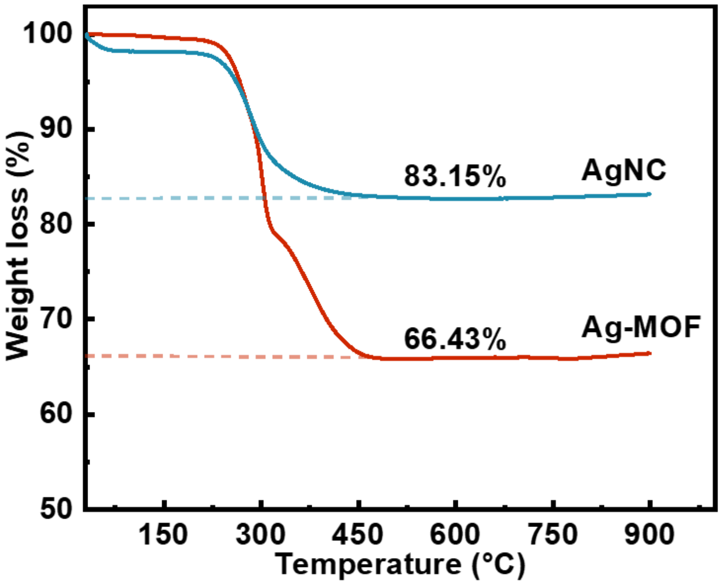

Figure S4. The TG curves of Ag-MOF and AgNC materials

The TG curves of Ag-MOF and AgNC were tested under air atmosphere with a heating rate of 10 °C min^{-1}, and the results are shown in Figure S4. For Ag-MOF and AgNC, the carbon substrate and organic linker will burn out to carbon dioxide in an air atmosphere, and the end product is silver metal. Based on the above analysis, the contents of Ag in AgNC and Ag-MOF were 83.15% and 66.43%, respectively. Further, the Ag content in Ag-MOF and AgNC was quantified as 50.30 wt% and 69.26 wt% by ICP-MS, respectively. The TG results showed that the Ag content in AgNC was higher than that in Ag-MOF, which was attributed to the fact that AgNC was prepared by high-temperature pyrolysis using Ag-MOF as the precursor. After pyrolysis, the highly dispersed molecular-level silver in Ag-MOF was severely agglomerated to form silver particles (Figure S5). Therefore, the silver content in AgNC is higher than that in Ag-MOF for the same mass.

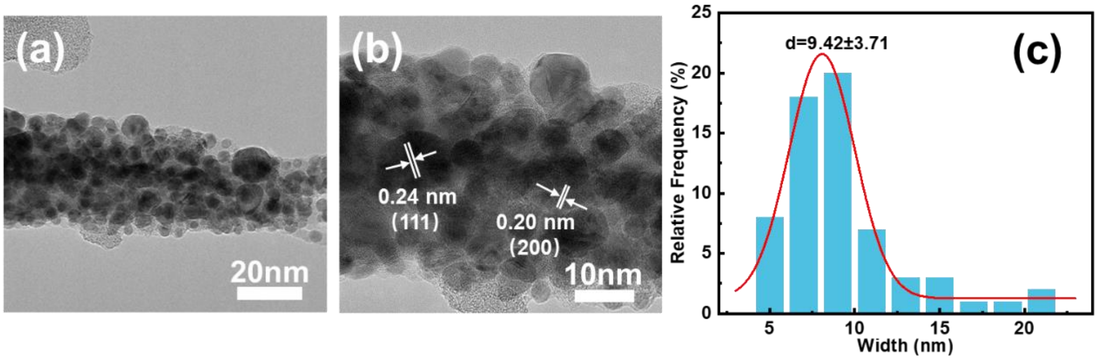

Figure S5. (a) TEM and (b) HRTEM images of AgNC, (d) corresponding the size distribution statistical histograms

As shown in Figure S5, the Ag nanoparticles in AgNC were agglomerated with a particle size of 9.42 nm compared to Ag-MOF. The HRTEM image shows a pair of well-defined lattice fringes, which correspond to the (111) and (200) crystal planes of the face-centered cubic Ag, respectively.

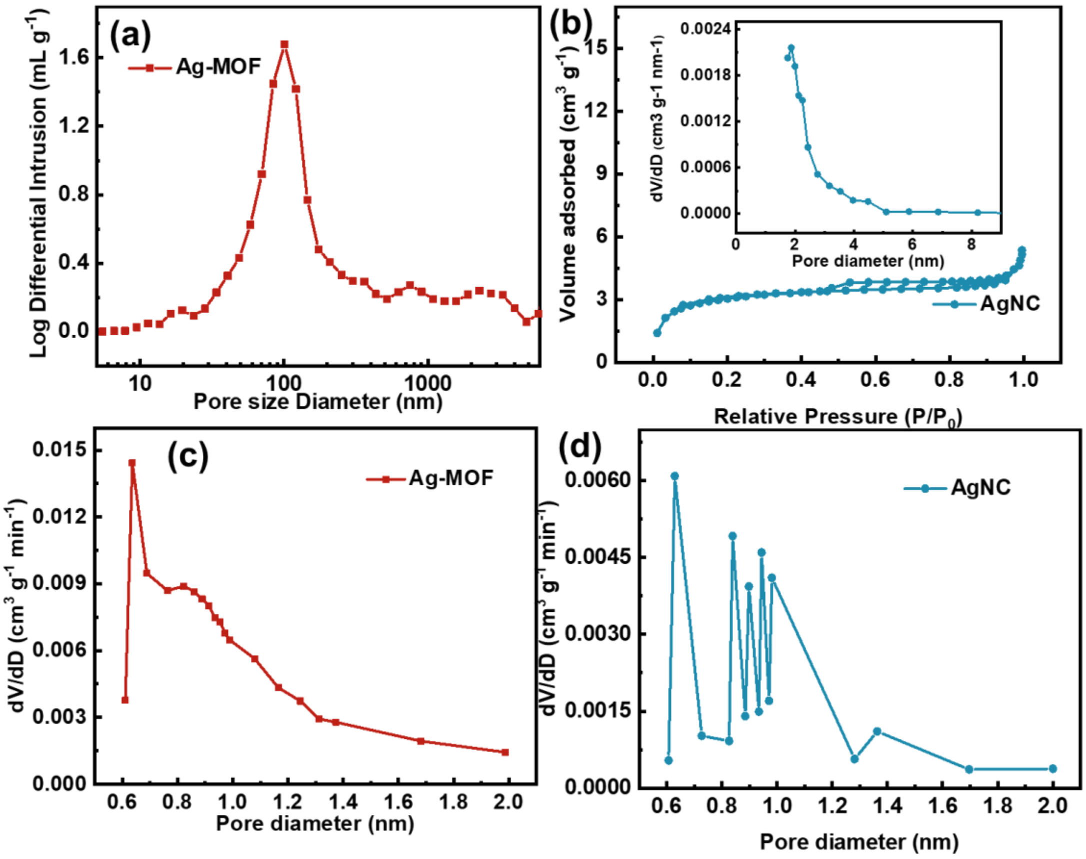

Figure S6. (a) The pore size distribution of Ag-MOF determined by mercury porosimetry. (b) N_2 adsorption-desorption isotherm of AgNC, and the inset is the corresponding pore size distribution curve. The microporous distribution determined by the HK method for (c) Ag-MOF and (d) AgNC.

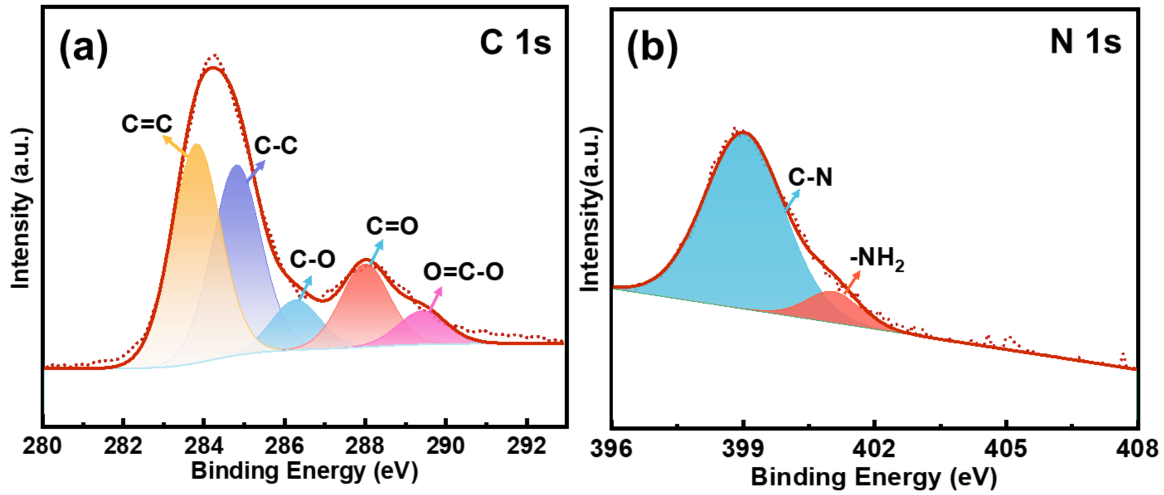

Figure S7. (a) XPS C 1s and (b) XPS N 1s spectra of Ag-MOF.

As shown in Figure S7a, the high-resolution C 1s spectra were fitted to five peaks, corresponding to C=C (283.79 eV), C-C (284.8 eV), C-O (286.29 eV), C=O (288.02 eV) and O=C-O (289.45 eV), respectively. The N 1s spectra is shown in Figure S7b, the peaks located at 398.99 eV and 401.05 eV are attributed to the C-N bond and $-NH_2$ group in the organic ligand, respectively.

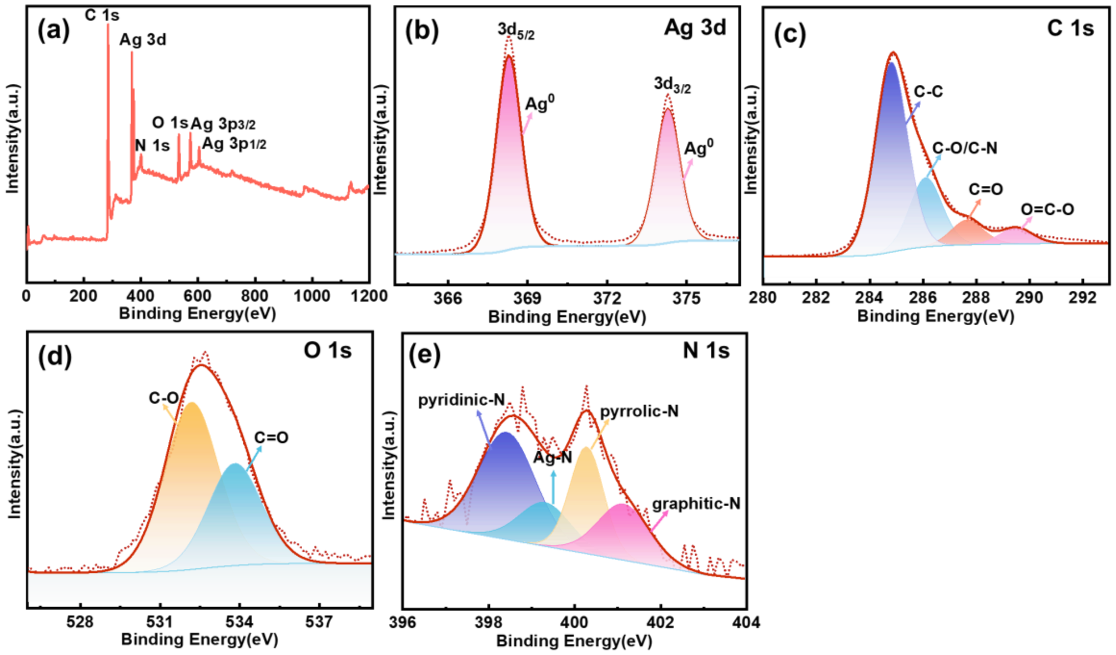

Figure S8. (a) XPS survey spectrum, (b) XPS Ag 3d, (c) Ni 2p, (c) XPS C 1s, (d) XPS O 1s and (f) XPS N 1s spectra of AgNC.

Figure S8 shows the analysis results of the XPS of AgNC. The XPS survey spectra of AgNC confirmed the presence of C, O, Ag and N elements (Figure S8a). The high-resolution Ag 3d spectra show a set of peaks with binding energies located at 368.29 and 347.29 eV, correlating to Ag $3d_{5/2}$ and Ag $3d_{3/2}$ of zero-valent Ag (Figure S8b), respectively. The Ag^+ present in Ag-MOF were completely reduced to metallic Ag due to carbothermal reduction. This is consistent with the XRD analysis. The high-resolution C1s spectra were fitted to four peaks, which were assigned to C-C (284.80 eV), C-O/C-N (286.11 eV), C=O (287.68 eV) and O=C-O (289.52 eV) peaks. The high-resolution O1s spectra showed two peaks identified as C-O (532.19 eV) and C=O (533.83 eV) bonds (Figure S8c). The high-resolution N 1s spectra were deconvoluted into four peaks, designated pyridine N (398.11 eV), Ag-N (399.06 eV), pyrrole N (400.61 eV) and graphite N (403.40 eV) (Figure S8d), respectively. The presence of nitrogen species is ascribed to the conversion of organic ligands to nitrogen-doped carbon in a pyrolysis process under silver catalysis.

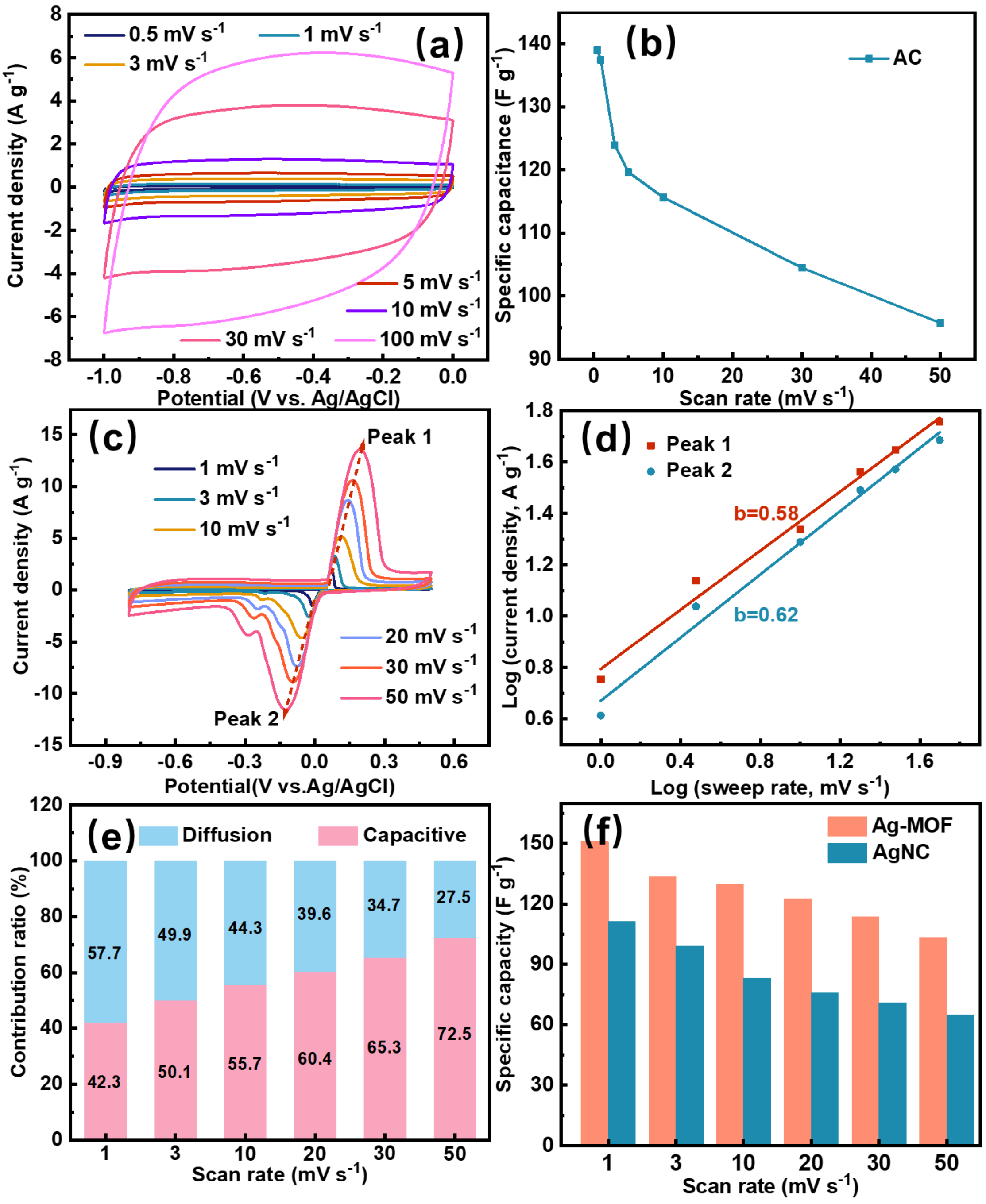

Figure S9. (a) The CV curves of AC at different scan rates, and (b) the specific capacitance at the corresponding scan rates. (c) CV curves at different scan rates, (d) b-value calculation, (e) capacity contribution at different scan rates for AgNC electrode. (f) Specific capacitance at different scan rates of Ag-MOF and AgNC electrodes.

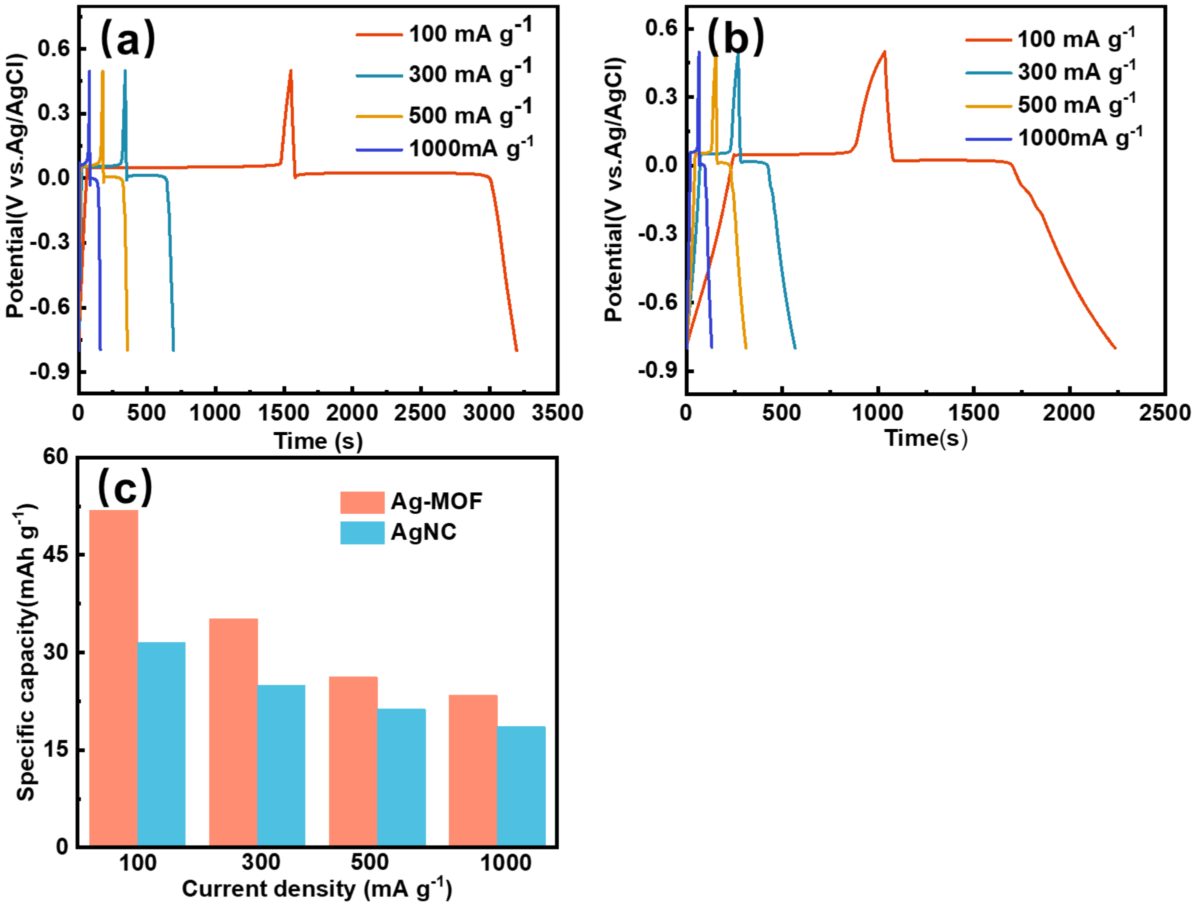

Figure S10. The GCD curves at different current densities: (c) Ag-MOF, (d) AgNC, and (e) the corresponding GCD specific capacity.

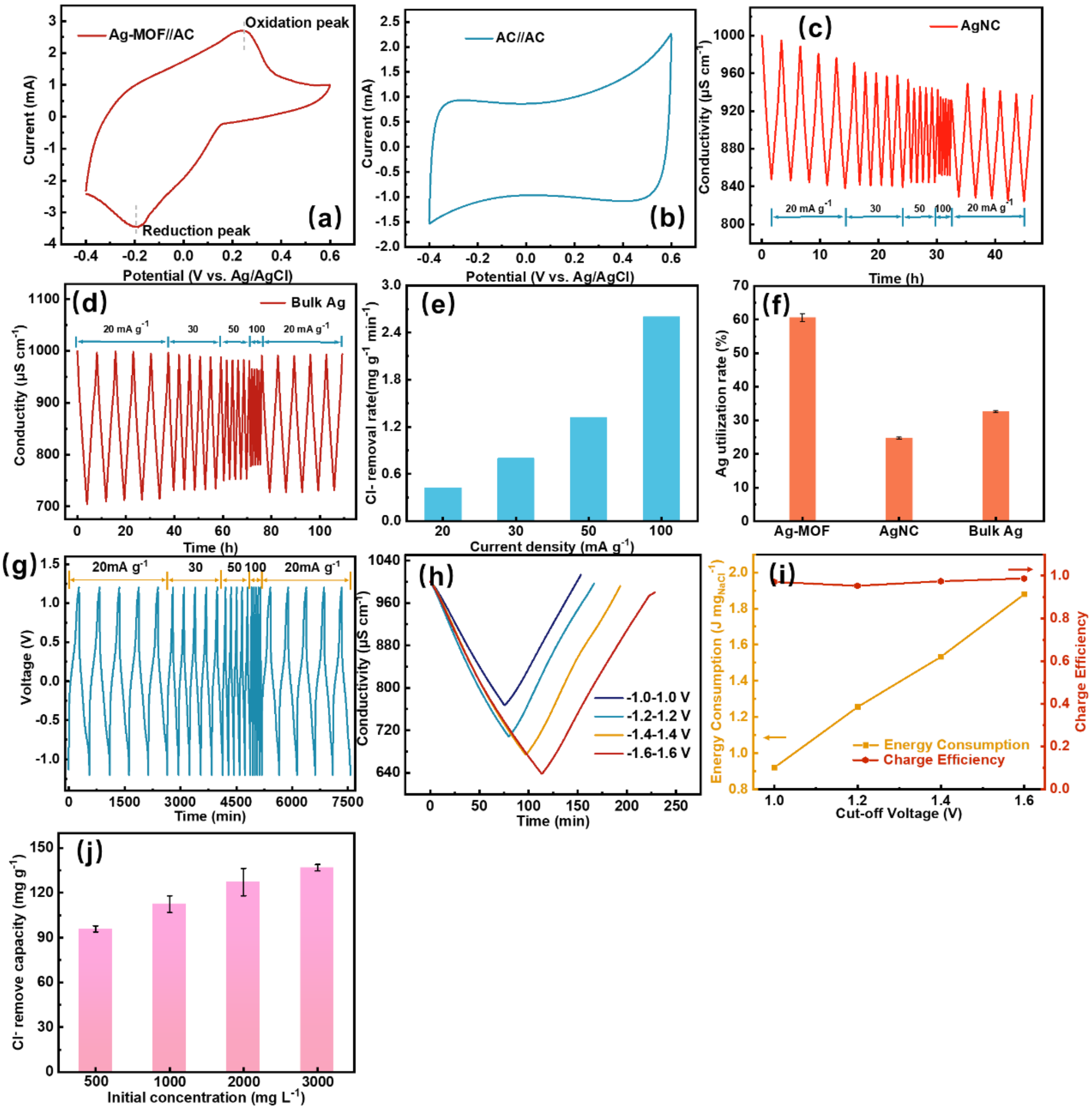

Figure S11. The CV curves for the CDI system: (a) Ag-MOF//AC, (b) AC//AC. The conductivity curves at different current densities for (c) AgNC and (d) Bulk Ag electrodes. (e) The Cl^- removal rate of Ag-MOF at different current densities with 500 mg L^{-1} NaCl solution. (f) The Ag utilization rate of Ag-MOF, AgNC and Bulk Ag electrodes during deionization. (g) The voltage versus time curves at different current densities, (h) the conductivity curves at different voltage windows with 500 mg L^{-1} NaCl solution, (i) the charge efficiency and energy consumption at different cutoff voltages, and (i) the Cl^- removal capacity in different initial NaCl solutions for Ag-MOF electrode.

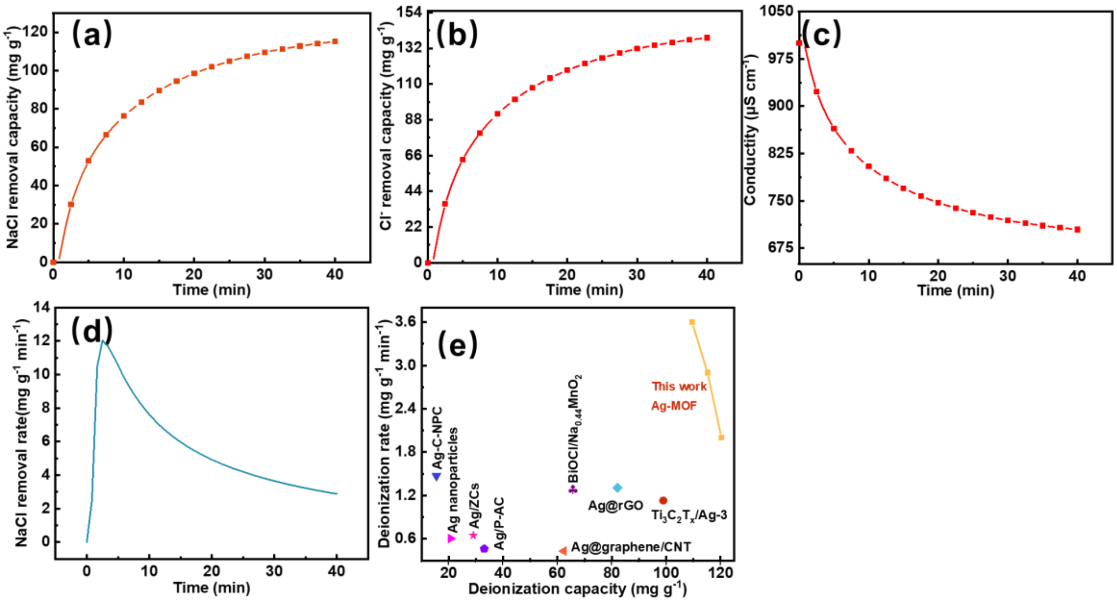

Figure S12. (a) Conductivity versus time curve, (b) NaCl concentration versus time curve, (c) Cl^- removal capacity versus time curve, and (d) NaCl removal rate versus time curve of Ag-MOF electrode during adsorption. (e) the performance comparison with previously reported Ag-based electrodes.

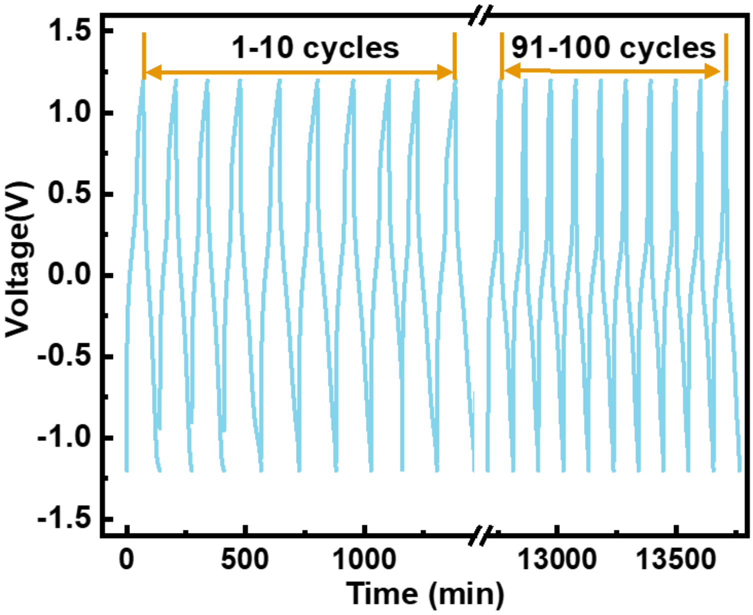

Figure S13. The voltage versus time curve of Ag-MOF electrode during 100 electrosorption cycles.

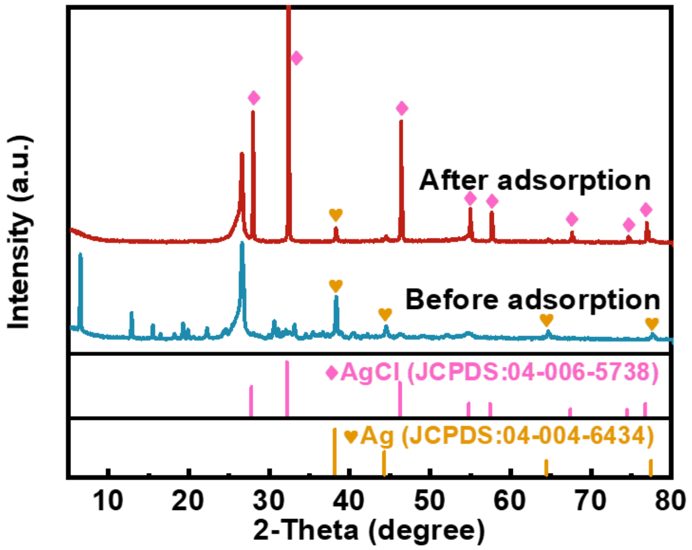

Figure S14. The XRD pattern of Ag-MOF electrode before and after adsorption in NaCl solution without applied voltage.

As shown in Figure 14, the XRD phase of the Ag-MOF electrode was almost completely converted to the peak of AgCl without applying voltage in 500 mg L^{-1} of NaCl solution for 40 min, and the peak of metal Ag^0 remained, confirming the presence of Ag^+ in the Ag-MOF structure.

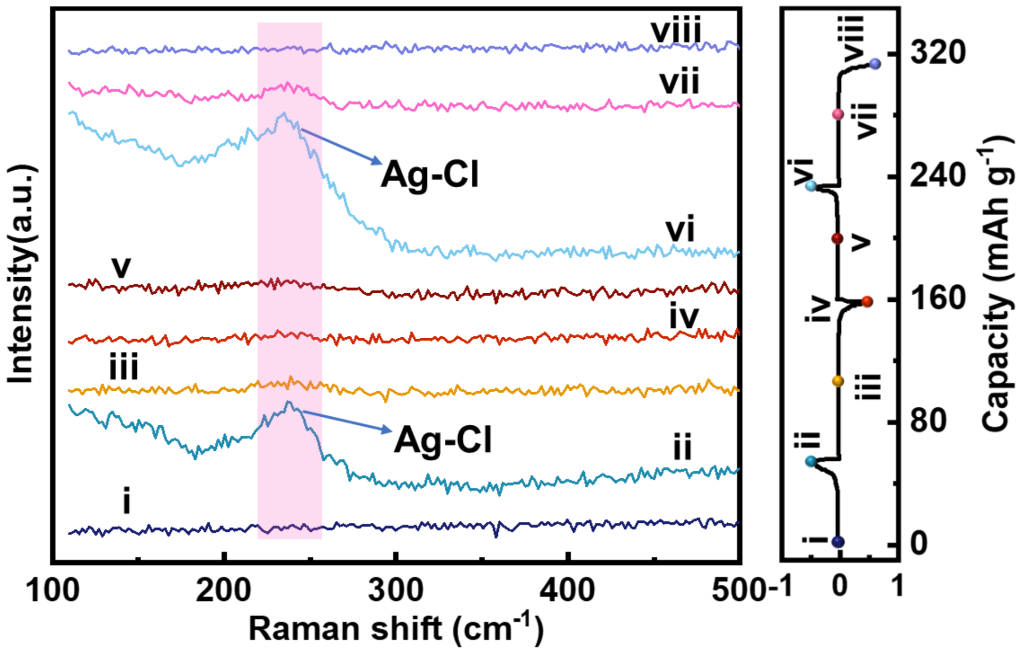

Figure S15. The Raman spectra of Ag-MOF electrodes in different charge and discharge states.

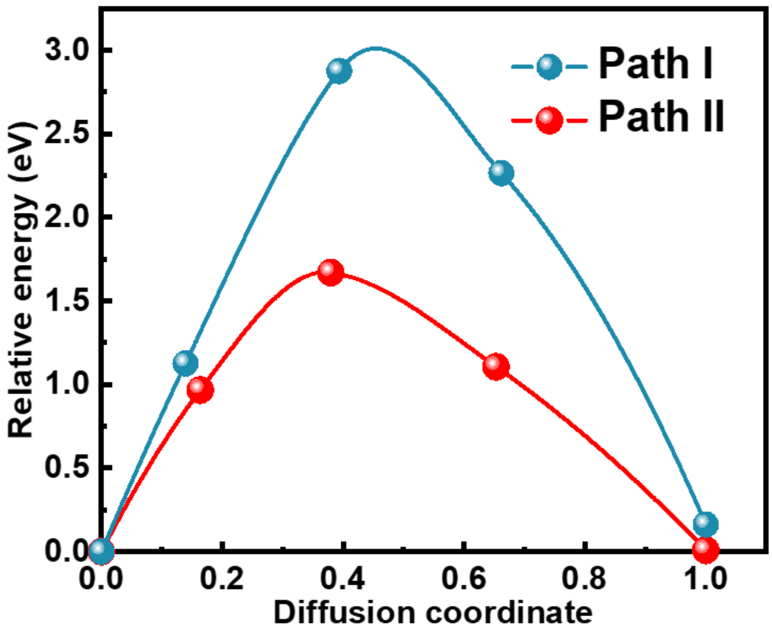

Figure S16. The diffusion energy barrier distribution of Cl^- for path I and path II in Ag-MOF.

Table S1. Surface texture properties of as-prepared samples.

**Samples**	**S_{BET} (m^2 g^{-1})**	**V_{tot} (cm^3 g^{-1})**	**V_{meso} (cm^3 g^{-1})**
Ag-MOF	85.23	0.087	0.085
AgNC	54.37	0.029	0.005

Table S2. Element content of various samples

Samples	C (wt%)	O (wt%)	Ag (wt%)	N (wt%)
Ag-MOF	63.49	20.77	9.72	6.02
AgNC	83.10	7.42	3.89	5.59

Table S3. Summary on the electrochemical performances of Ag-MOF and AgNC.

Samples	Ag-MOF	AgNC
R_s/Ω	3.95	1.83
R_{ct}/Ω	1.85	1.97

Table S4. Comparative results on the removal capacity among different materials.

Materials	Voltage/ Current Density	NaCl concentration (mg L^{-1})	Cl^- removal capacity (mg g^{-1})	References
Core-shell CoFe-LDH	1.2 V	600	41.51	1
ZnCo-Cl LDH	1.2 V	500	66.03	2
Chloride pre-intercalated CoFe-LDH	150 mA g^{-1}	1170	60.81	3
CuAl-LDO	1.2 V	500	23.72	4
Calcined MgAl-LDH/Graphene	1.0 V	500	16.51	5
Cl-FeOOH	1.2 V	500	42.62	6
Ag-coated activated carbon	1.2 V	5850	26.51	7
Ag/N-CNP	1.2 V	824	75.30	8
Ag-decorated monolithic	1.5 V	824	68.74	9

carbon sponge				
Ag/ZCs	1.2 V	100	24.04	10
Commercial Bi powder	1.2 V	500	33.73	11
$NaBi_3O_4Cl_2$	60 mA g^{-1}	29250	58.40	12
Ag-MOF	20 mA g^{-1}	500	121.52	This work
Ag-MOF	1.2 V	500	115.16	This work

■ References

(1) Li, Z.; Mao, S.; Yang, Y.; Sun, Z.; Zhao, R. Controllable Synthesis of a Hollow Core-Shell Co-Fe Layered Double Hydroxide Derived from Co-MOF and Its Application in Capacitive Deionization. *J Colloid Interface Sci* **2021**, *585*, 85–94. https://doi.org/10.1016/j.jcis.2020.11.091.

(2) Zhang, Z.; Li, H. Promoting the Uptake of Chloride Ions by ZnCo–Cl Layered Double Hydroxide Electrodes for Enhanced Capacitive Deionization. *Environ Sci Nano* **2021**, *8* (7), 1886–1895. https://doi.org/10.1039/D1EN00350J.

(3) Wang, K.; Liu, Y.; Ding, Z.; Chen, Z.; Xu, X.; Wang, M.; Lu, T.; Pan, L. Chloride Pre-Intercalated CoFe-Layered Double Hydroxide as Chloride Ion Capturing Electrode for Capacitive Deionization. *Chemical Engineering Journal* **2022**, *433*, 133578. https://doi.org/10.1016/j.cej.2021.133578.

(4) Xi, W.; Li, H. Vertically-Aligned Growth of CuAl-Layered Double Oxides on Reduced Graphene Oxide for Hybrid Capacitive Deionization with Superior Performance. *Environ Sci Nano* **2020**, *7* (3), 764–772. https://doi.org/10.1039/C9EN01238A.

(5) Ren, Q.; Wang, G.; Wu, T.; He, X.; Wang, J.; Yang, J.; Yu, C.; Qiu, J. Calcined MgAl-Layered Double Hydroxide/Graphene Hybrids for Capacitive Deionization. *Ind Eng Chem Res* **2018**, *57* (18), 6417–6425. https://doi.org/10.1021/acs.iecr.7b04983.

(6) Zhao, J.; Wu, B.; Huang, X.; Sun, Y.; Zhao, Z.; Ye, M.; Wen, X. Efficient and Durable Sodium, Chloride-doped Iron Oxide-Hydroxide Nanohybrid-Promoted Capacitive Deionization of Saline Water via Synergetic Pseudocapacitive Process. *Advanced Science* **2022**, *9* (25), 2201678. https://doi.org/10.1002/advs.202201678.

(7) Yoon, H.; Lee, J.; Min, T.; Lee, G.; Oh, M. High Performance Hybrid Capacitive Deionization with a Ag-Coated Activated Carbon Electrode. *Environ Sci (Camb)* **2021**, *7* (7), 1315–1321. https://doi.org/10.1039/d1ew00209k.

(8) He, Y.; Huang, L.; Zhao, Y.; Yang, W.; Hao, T.; Wu, B.; Deng, H.; Wei, D.; Wang,

H.; Luo, J. A Newly Synthesized Highly Stable Ag/N-Carbon Electrode for Enhanced Desalination by Capacitive Deionization. *Environ Sci Nano* **2020**, *7* (10), 3007–3019. https://doi.org/10.1039/d0en00826e.

(9) Yan, L.; Wu, B.; Zhao, Y.; Annor Asare, J.; Gang, H.; Wei, D.; Cao, Y.; Chen, P.; Wang, H.; Huang, L. Ag-Decorated Monolithic Carbon Sponge with Porous Directional Frameworks for Chloride Removal in Flow-through Capacitive Deionization. *Sep Purif Technol* **2023**, *322*. https://doi.org/10.1016/j.seppur.2023.124210.

(10) Zhang, H.; Zhang, W.; Shen, J.; Li, Y.; Yan, X.; Qi, J.; Sun, X.; Shen, J.; Han, W.; Wang, L.; Li, J. Ag-Doped Hollow ZIFs-Derived Nanoporous Carbon for Efficient Hybrid Capacitive Deionization. *Desalination* **2020**, *473*. https://doi.org/10.1016/j.desal.2019.114173.

(11) Chang, J.; Duan, F.; Su, C.; Li, Y.; Cao, H. Removal of Chloride Ions Using a Bismuth Electrode in Capacitive Deionization (CDI). *Environ Sci (Camb)* **2020**, *6* (2), 373–382. https://doi.org/10.1039/c9ew00985j.

(12) Wei, W.; Feng, X.; Wang, R.; Zheng, R.; Yang, D.; Chen, H. Electrochemical Driven Phase Segregation Enabled Dual-Ion Removal Battery Deionization Electrode. *Nano Lett* **2021**, *21* (11), 4830–4837. https://doi.org/10.1021/acs.nanolett.1c01487.
